# Mechanism of Action and Structure–Activity Relationships of Tetracyclic Small Molecules Acting as Universal Positive Allosteric Modulators of the Cholecystokinin Receptor

**DOI:** 10.3390/membranes13020150

**Published:** 2023-01-24

**Authors:** Daniela G. Dengler, Kaleeckal G. Harikumar, Alice Yen, Eduard A. Sergienko, Laurence J. Miller

**Affiliations:** 1 Conrad Prebys Center for Chemical Genomics, Sanford Burnham Prebys Medical Discovery Institute, La Jolla, CA 92037, USA; 2 Department of Molecular Pharmacology and Experimental Therapeutics, Mayo Clinic, Scottsdale, AZ 85259, USA

**Keywords:** cholecystokinin, cholecystokinin receptor, G protein-coupled receptor, positive allosteric modulator, obesity

## Abstract

As part of an ongoing effort to develop a drug targeting the type 1 cholecystokinin receptor (CCK1R) to help prevent and/or treat obesity, we recently performed a high throughput screening effort of small molecules seeking candidates that enhanced the action of the natural agonist, CCK, thus acting as positive allosteric modulators without exhibiting intrinsic agonist action. Such probes would be expected to act in a temporally finite way to enhance CCK action to induce satiety during and after a meal and potentially even modulate activity at the CCK1R in a high cholesterol environment present in some obese patients. The current work focuses on the best scaffold, representing tetracyclic molecules identified through high throughput screening we previously reported. Extensive characterization of the two top “hits” from the previous effort demonstrated them to fulfill the desired pharmacologic profile. We undertook analog-by-catalog expansion of this scaffold using 65 commercially available analogs. In this effort, we were able to eliminate an off-target effect observed for this scaffold while retaining its activity as a positive allosteric modulator of CCK1R in both normal and high cholesterol membrane environments. These insights should be useful in the rational medicinal chemical enhancement of this scaffold and in the future development of candidates to advance to pre-clinical proof-of-concept and to clinical trials.

## 1. Introduction

The type 1 cholecystokinin receptor (CCK1R) has been recognized as a key physiologic regulator of appetite and a potential target for anti-obesity therapy [1]. However, while multiple full agonists of CK1R have been developed, these molecules have failed to achieve the primary endpoint in clinical obesity trials of being superior to short-term dieting to induce weight loss [2,3,4]. The enhancement of potency and/or duration of action of such candidate molecules has not been pursued due to concerns about on-target side effects and potential toxicity [3,5]. We recently reported a high throughput screening effort to identify potential molecules with a distinct pharmacologic profile representing positive allosteric modulators (PAMs) of CCK action at this receptor that also possess minimal intrinsic agonist activity [6], a strategy we proposed to increase the safety and effectiveness of such drugs [3,5]. We were also particularly interested in identifying such compounds that were active both in a normal and high cholesterol membrane environment representing universal PAMs. Of the three chemical classes of molecules identified in that effort [6], we focused on those molecules from the initial high throughput screening effort that incorporate a tetracyclic scaffold (“hit 1” and “hit 6”) as being the most promising. 

In the present project, we explore the molecular basis of action of this class of CCK1R PAM ligands and expand our understanding of structure–activity relationships of molecules with this tetracyclic scaffold, exploring structural modifications present in 65 commercially available analogs. Since our preliminary work with the original “hits” having this scaffold demonstrated an effect on another class A G protein-coupled receptor, the vasopressin 2 receptor, a parallel goal was to establish the feasibility of teasing apart on-target and off-target activity as an indication of the general selectivity of this scaffold among this family of receptors. Such insights will be useful to guide further rational enhancement of the pharmacologic activity profile and development of drug candidates to advance toward future preclinical and potential clinical trials. 

## 2. Materials and Methods

### 2.1. Materials

CCK peptide analogues were custom synthesized in our laboratory, purified to homogeneity, and verified by mass spectrometry [7]. These include natural CCK-26-33 (CCK-8); a partial agonist analogue, D-Tyr-Gly-[(Nle^28,31^)CCK-26-32]-phenethyl ester (CCK-OPE); and a fluorescent analogue of this hormone, alexa488-D-Tyr-Gly-[(Nle^28,31^)CCK-26-33] (alexa488-CCK) [8]. The sulfated CCK octapeptide (CCK-8) (#4033010) used in the structure–activity relationship studies and arginine vasopressin (#4012215) were purchased from Bachem AG (Bubendorf, Switzerland). CCK-33 was purchased from Peptides International (Louisville, KY, USA). Clonal receptor-bearing cell lines were prepared from non-CCK receptor-bearing CHO-K1 cells or HEK-293 cells (American Type Culture Collection, ATCC), as previously described [9]. In select experiments, the cholesterol composition of cell lines was enhanced by treatment with methyl-β-cyclodextrin-cholesterol inclusion complex, as we previously described [10]. 

### 2.2. Methods

Biological activity was quantified in the CHO cell lines described above using intracellular calcium assays that were performed, as previously described, using Fura-8-AM (AAT Bioquest, Pleasonton, CA, USA) [11]. Full agonist dose–response curves were performed along with determination of maximal responses to 0.1 mM ATP, targeting an endogenous receptor present on the parental cells. Concentration–response curves of peak intracellular calcium responses were analyzed and plotted as percentages of maximal responses using non-linear regression analysis in Prism 9.1 (GraphPad). 

For structure–activity relationship studies, we performed myo-inositol-1-phosphate (IP-One) accumulation assays with TR-FRET technology applied to the HEK-293 cell line overexpressing CCK1Rs, as we had described in our high throughput screening effort [6]. In brief, thawed cryopreserved cell stocks were re-suspended in IP-One assay media consisting of phenol-red free DMEM (Corning, Corning, NY, USA, #17-205-CV) with 10% FBS, 1% penicillin (10,000 units)/streptomycin (10 mg) (Pen/Strep, Thermo Fisher Scientific, Waltham, MA, USA, Gibco #15140122), and 1% L-glutamine (200 mM) (Gibco #25030081) and diluted to required cell densities. Then, the cell suspension was dispensed into a 1536-well tissue culture microplate (Corning, #3727), and the plate was incubated overnight at 37 °C and 5% CO^2^. After 16–20 h, DMSO or compounds were added with an Echo liquid handler (Labcyte, San Jose, CA, USA), resulting in a top final compound concentration of 50 µM diluted 2-fold for 16-point dose–responses. For the positive allosteric modulator (PAM) format, orthosteric stimulator (CCK) dilutions for control wells were prepared in assay media containing 150 mM lithium chloride (LiCl, 50 mM final, Sigma-Aldrich, St Louis, MO, USA, #L7026). After a 30 min incubation of test compounds at 37 °C and 5% CO_2_, orthosteric stimulator dilutions (for PAM format) or equivalent dilutions of DMSO (for agonist format) were added to designated wells using BioRaptr (Beckman Coulter, Brea, CA, USA). In addition, CCK control ligand dilutions were included on each test plate. After that, the plate was incubated for 1 h at 37 °C and 5% CO^2^ and equilibrated to room temperature. Detection reagents from the IP-One Gq detection kit from Cisbio (Cisbio US Inc., Bedford, MA, USA, #62IPAPEJ) were added, and after 1 h at RT, IP-One content was measured with a PHERAstar FSX microplate reader (BMG Labtech, Ortenberg, Germany). Control ligand dose–responses were analyzed using GraphPad Prism 9.3.1 (San Diego, CA, USA) to validate adequate control and test well concentrations for positive allosteric modulation (PAM) and agonist formats. Dose–response curves were performed as 16-point 2-fold dilutions in duplicates in at least three independent experiments. Large data sets were uploaded and analyzed with CBIS (Chemical and Biology Information System software, ChemInnovation Software, Inc., San Diego, CA, USA). Further data analysis for detailed SAR studies was conducted using the D360 software (Certara). 

Possible off-target biological effects. In our previous report [6], the lead compounds were tested for possible off-target effects at the purinergic receptor (P2YR), representing another Gq-coupled class A GPCR, where they were found to have no off-target activity. Here, we tested them at the vasopressin-2 receptor (AVP2R), representing a class A GPCR structurally related to CCK1R, which is coupled to Gs. The activity at AVP2R was tested using AVP2R-overexpressing CHO-K1 (PAM format) or HEK-293 (agonist format) cells, stimulating the cells with vasopressin. Original hits were tested in both agonist and PAM formats. Since all recognized compound responses were present in both formats, we continued to screen analogs in only the PAM format (also able to capture agonist effects) in CHO-K1 AVP2R cells for potential enhanced sensitivity. The dose–response data sets for original hits were combined, as the PAM and agonist effects were indistinguishable. In brief, arginine vasopressin (AVP) orthosteric stimulator dilutions and ligand and compound dose–response titrations in DMSO were transferred onto a 1536-well plate (Corning #3725) using an Echo liquid handler (Labcyte). Thawed cryopreserved cell stocks were diluted in stimulation buffer consisting of HBSS (Hank’s Balanced Salt Solution with Ca^2+^ and Mg^2+^, Gibco #24020117), 5 mM HEPES (hydroxyethyl piperazineethanesulfonic acid), 0.5 mM IBMX (3-isobutyl-1-methylxanthine, Sigma-Aldrich), and 0.075% BSA (7.5% DTPA-purified bovine serum albumin, PerkinElmer). The resulting cell suspension was added to the microplate and incubated with test compounds at room temperature for 30 min. Then, the detection reagents from the Cisbio cAMP Gs Dynamic HTRF detection kit were added. The plate was kept at room temperature for 30 min and read on a PHERAstar FSX microplate reader. Dose–response curves were performed as 16-point 2-fold dilutions in duplicate in at least two independent experiments. 

For CCK1R-induced Gs signaling studies, we performed cAMP accumulation assays as described above and as previously reported [6], utilizing the HEK-293 cells overexpressing CCK1Rs and the EC_20_ concentration of CCK for screening in PAM format.

Regarding CCK binding kinetics, fluorescence polarization assays for binding and dissociation of the fluorescent CCK probe, alexa488-CCK, were performed as we described using a PHERAstar FSX instrument (BMG Labtech, Cary, NC, USA) [12]. 

Receptor internalization assays were performed with the fluorescent alexa488-CCK ligand used to visualize cell surface receptors after treatment and fixation with 2% paraformaldehyde (Electron Microscopy Sciences, Cat# 15710), as we described [12]. Images were acquired with a Zeiss Axiovert 200M inverted epifluorescence microscope. 

For structure–activity analysis, 65 commercially available compounds containing the tetracyclic scaffold were included in this analysis to gain insights into structure–activity relationships (structures shown in SAR tables). 

The potency and efficacy data for these compounds were used to calculate their activity scores using the following formula: Activity Score = normalized E_max_ × pEC_50_. Normalized E_max_ represents the efficacy of the compound as a fractional response relative to that of the CCK control ligand. pEC_50_ represents the positive logarithmic value of compound EC_50_ concentrations in (M).

Because this was not a systematic prospective synthetic series, compounds often modified more than one position around the tetracyclic scaffold at a time. We elected to number the entire list of analogues in order from highest to lowest activity score (described below) in the primary screen, the PAM format CCK-stimulated IP-One assay (CMPs 1-65). In total, 37 of these compounds had a measurable activity score in this assay. We grouped the compounds in six SAR tables to focus on groups of related structures, and we ordered each group of structures from most to least active in the primary assay when possible. By comparing the position in each table and the compound number, it was clear when multiple modifications may have played a role in the series. These compounds were also characterized with the off-target AVP2R assay described above, with results listed in the same tables. 

Regarding statistical analysis, all assays were performed in duplicate and repeated in at least three independent assays (number of such assays, “*n*” provided). Differences between experimental groups were evaluated using one-way ANOVA or the Mann–Whitney test, with *p* < 0.05 considered as significant.

## 3. Results

**Figure 1:**
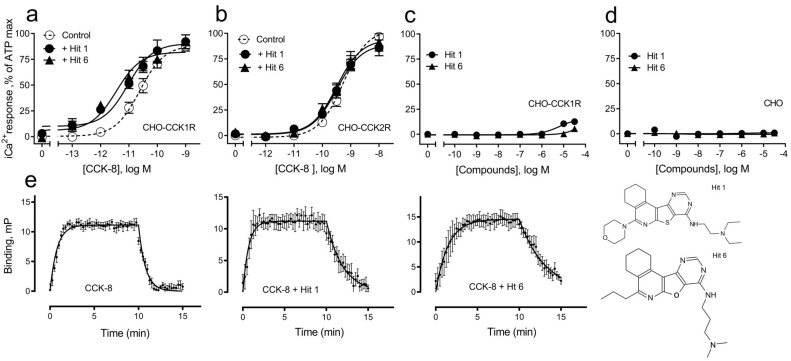
Pharmacologic profiles of “hits” 1 and 6. Shown are the structures of these two ligands, as well as evidence for their positive allosteric modulation of CCK action to stimulate intracellular calcium using Fura-8-AM at the CCK1R expressed on the CHO-CCK1R cell line (**a**), with no effect on CCK2R in the analogous CHO-CCK2R cell line (**b**) and no significant endogenous agonist activity at CCK1R in the CHO-CCK1R cell line (**c**) or at parental CHO cells (**d**). “Hits” 1 and 6 were utilized in 10 µM concentrations. Maximal intracellular calcium responses were determined using 0.1 mM ATP targeting an endogenous CHO cell receptor. Values represent means ± SEM of data from a minimum of 5 independent experiments performed in duplicate and are analyzed in Table 1. Both ligands exerted their positive allosteric modulatory effect on CCK action at CCK1R by prolonging the peptide off rate (**e**). Kinetic data represent 5 independent experiments, with results analyzed in Table 2.

The two hits initially identified in our high throughput screen for positive allosteric modulators (PAMs) of CCK action at the CCK1R possessing minimal intrinsic agonist activity [6] were further characterized to gain insights into their mechanism of action. Both of these compounds possess the same tetracyclic scaffold (structures shown in Figure 1). Figure 1 also shows the ability of these compounds to shift the CCK-8 concentration–response curve for stimulating intracellular calcium in CCK1R to the left, reflecting their PAM activity (Figure 1a and Table 1). This is unique to its action at CCK1R, with no analogous impact on CCK activity at CCK2R (Figure 1b and Table 1). Neither compound had any demonstrable, statistically significant intrinsic agonist action in concentrations as high as 20 µM at the CCK1R over-expressing CHO cell line (*p* = 0.1) (Figure 1c), and neither compound exhibited any demonstrable agonist activity at the parental CHO cells, even at 20 µM concentrations (Figure 1d). A longer molecular form of CCK, CCK-33, also had its biological effect augmented significantly by both of these compounds (Table 1).

The PAM activity of these compounds was explained by their slower dissociation rate for CCK, shown in Figure 1e, resulting in prolonged receptor occupation times. The kinetic parameters are shown in Table 2.

**Figure 2:**
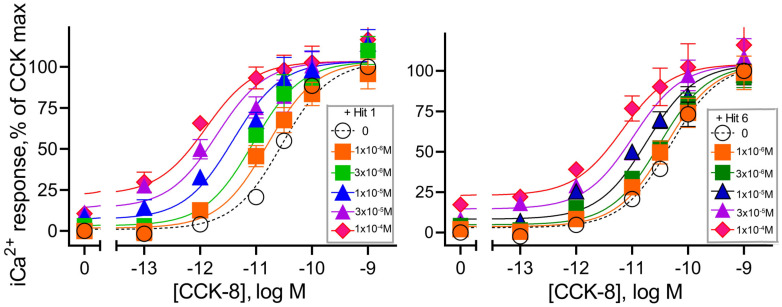
Positive allosteric modulation of CCK action at CCK1R expressed on CHO-CCK1R cells. Shown are the abilities of “hits” 1 and 6 utilized in 10 µM concentrations to shift the CCK concentration–response curves to the left in a dose-dependent manner. Values were plotted as percentages of maximal intracellular calcium responses to CCK in each replicate curve. There were no statistically significant differences in the maximal responses achieved under any condition. Data plotted represent means ± SEM and are analyzed in Table 3.

The allosteric constants for these compounds were calculated based on their concentration–response curves for enhancing CCK-stimulated intracellular calcium responses in the CCK1R-expressing cells (Figure 2). Using the operational model for allosterism (Prism 9.1, GraphPad), we determined log(αβ) values of 1.5 and 1.0 for hit 1 and hit 6, respectively (Table 3), which confirmed their positive cooperativity with natural CCK peptide at CCK1Rs. 

**Figure 3:**
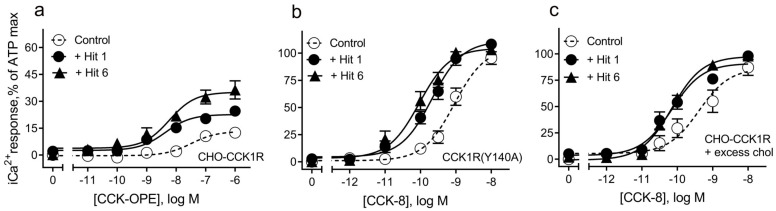
Activity of “hits” 1 and 6 on partial agonist activity and CCK activity in CHO cell lines expressing the noted receptor constructs. “Hits” 1 and 6 were utilized in 10 µM concentrations. Shown is the ability of these ligands to affect the intracellular calcium activity measured with Fura-8-AM of a partial agonist of CCK1R and CCK-OPE (**a**), and their ability to act as positive allosteric modulators of CCK action at a mimic of the high cholesterol state of CCK1R and CCK1R(Y140A) (**b**) and at CCK1R in a high cholesterol environment (**c**). Values represent means ± SEM of data from 5 independent experiments performed in duplicate.

Part of the rationale for developing these compounds relates to the possibility of correcting the aberrant stimulus–activity coupling observed at the CCK1R in a membrane environment with high cholesterol, as sometimes seen in obesity. Figure 3 shows the ability of both compounds to exhibit PAM action of a partial agonist acting at CCK1R and CCK-OPE (Figure 3a), as well as at the CCK1R(Y140A) receptor construct known to mimic CCK1R in high cholesterol [11] (Figure 3b), and at wild type CCK1R in the setting of elevated cholesterol (Figure 3c). The rationale for using this partial agonist was to attempt to amplify the PAM activity even though this is not a physiologic ligand. Indeed, using CCK-OPE, we observed not only a left shift in the concentration–response curves, but also increases in maximal responses. This continues to be quite encouraging and to further fulfill the pharmacologic profile of interest. 

**Figure 4:**
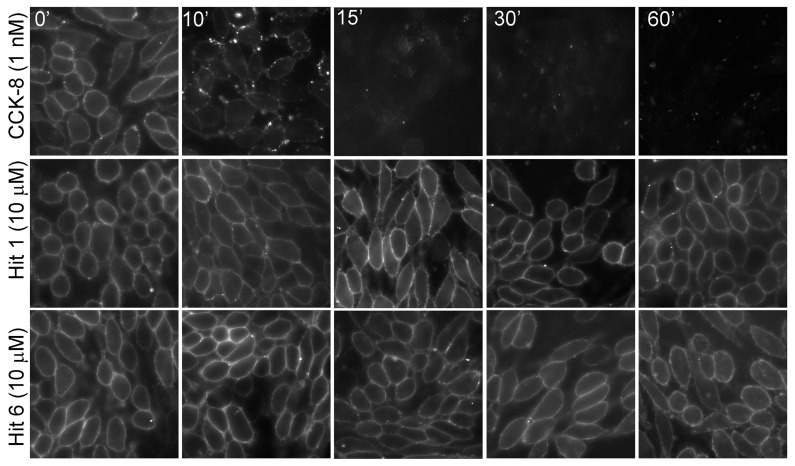
Impact of “hits” 1 and 6 on CCK1R internalization. “Hits” 1 and 6 were utilized in 10 µM concentrations. Alexa488-CCK was utilized to label the cell surface CCK receptor. Shown are time-dependent fluorescence images of cell surface CCK1R after exposure to CCK and the two “hits”. CCK stimulated prompt receptor internalization, while neither “hit” cleared the receptor from the cell surface. Images shown are representative of data from three independent experiments.

For a small molecule to modulate the action of endogenous CCK, it is important that it occupies the receptor while on the cell surface and does not stimulate its internalization. The receptor is therefore primed for its enhanced response to endogenous CCK when released after a meal. Figure 4 shows that these compounds when used in concentrations as high as 10 µM did not stimulate CCK1R internalization. 

To determine potential off-target effects, we screened the original hits, “hit 1” (CMP-1) and “hit 6” (CMP-28), against other GPCRs, including P2YRs [6] and vasopressin-2 receptors (AVP2Rs). Neither compound exhibited any activity at the P2YR. We found that CMP-1 (EC_50_ 12 μM, E_max_ 21%) and particularly CMP-28 (EC_50_ 15 μM, E_max_ 73%) showed significant activity in AVP2R cAMP assays in both agonist and PAM formats (combined data). Since the PAM format captures both the agonist and PAM responses with comparable sensitivity, we included this assay as part of our effort to characterize structure–activity relationships (SARs) of the tetracyclic scaffold to identify structural determinants that could eliminate AVP2R activity while maintaining or increasing PAM effects at CCK1R.

**Figure 5:**
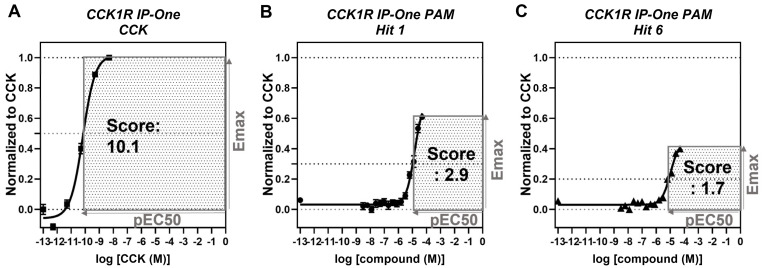
Activity score represents an approximation of the AUC of compound dose–response curves. IP-One dose–response of (**A**) CCK-8, (**B**) “hit 1”, and (**C**) “hit 6” in HEK-293 CCK1R cells; TR-FRET ratios resulting from IP-One accumulation normalized to CCK (E_max_ = 1) with (**A**) DMSO or (**B**,**C**) EC_20_ CCK as negative control (E_max_ = 0), representing (**A**) agonist and (**B**,**C**) PAM screening format, respectively; activity score was calculated by multiplying pEC_50_ with normalized E_max_, and the corresponding area is highlighted in grey; graphs plotted using GraphPad Prism; experiments performed in duplicate in at least three independent experiments; data points shown as mean ± SEM.

In exploring SARs of the tetracyclic scaffold, seeking PAMs, the CCK1R IP-One assay performed in PAM mode provided EC_50_ and E_max_ values, reflecting both potency and efficacy. Cooperative effects on either of these parameters could contribute to the desired PAM impact. We therefore utilized a composite activity score (multiplying pEC_50_ and E_max_ normalized to the maximal response to the control compound, CCK) to help prioritize the data. This score represents an approximation of the area under the curve (AUC) of a compound dose–response curve using normalized data respective the screening format (Figure 5). We found that combining EC_50_ and E_max_ into one score facilitated the comparison of PAMs. We determined an activity score of 2.9 for “hit 1” and a score of 1.7 for “hit 6” and found these scores to be more representative of the allosteric activities (log(αβ) 1.5 (hit 1) and 1.0 (hit 6)) than focusing separately on potencies or efficacies. For comparison, the activity score of CCK in the CCK1R IP-One assay was calculated at 10.1. Hence, the AUC of “hit 1” was approximately 29% of the AUC of the endogenous agonist CCK. In general, we found that activity scores below 1.5 represented non-significant activation in the tested format. Shown in Table 4, Table 5, Table 6, Table 7, Table 8 and Table 9 are the data from the CCK1R IP-One PAM screening for the 65 compounds representing analogs built on the tetracyclic scaffold of interest. These have been numbered based on descending order of PAM activity scores. 

**Figure 6:**
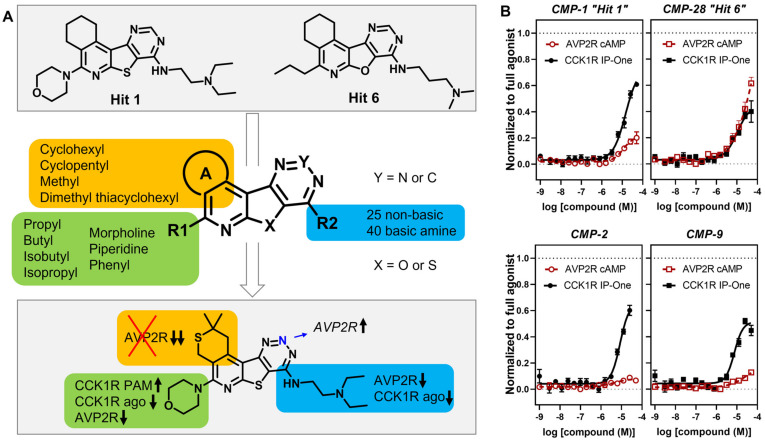
Elucidation of structural determinants impacting the pharmacological profile of tetracyclic analogs (**A**). “Hits” 1 and 6 were subjected to a comprehensive SAR campaign elucidating structural features with distinct effects on CCK1R PAM, CCK1R agonist and AVP2R off-target activity. (**B**) Dose–response curves of “hit 1” and “hit 6” (top) and optimized dimethyl thiacyclohexyl analogs CMP-2 and CMP-9 (bottom) in CCK1R IP-One and AVP2R cAMP assays, both conducted in PAM format. Data are shown in Table 4, Table 5, Table 6, Table 7, Table 8 and Table 9.

An examination of the structures of “hit 1” (CMP-1) and “hit 6” (CMP-28) identified five sites amenable for chemical modifications, as depicted in Figure 6. We recognized that both hits contained a basic tertiary amine attached to the tetracyclic core through a diamino alkyl linker at the position marked as R2 with predicted pKas values of 9.2 and 9.7, respectively. In our previous report [6], we hypothesized that this basic amine might play a crucial role in the PAM activity of our hits. To further explore this hypothesis, we examined 25 analogs with non-basic substituents at R2 attached to the pyrimidine of the tetracyclic core (Table 4 and Table 5). 

With the aim to develop PAMs with no or minimal intrinsic agonist activity at CCK1Rs and no off-target effects, the compounds were profiled in a 16-point dose–response format in CCK1R IP-One PAM and agonist formats, as well as in AVP2R cAMP assays. We tested 15 direct analogs of “hit 1” with sulfur in the tetracyclic core, a cyclohexyl group as saturated ring A, and morpholine as residue R1. The results are listed in Table 4. 

To explore whether the basic amine is part of an ionic interaction or rather acts via hydrogen bonds with the receptor, we investigated analogs with a methoxy (CMP-47) or hydroxy (CMP-49) function instead of the diethyl amine group, as well as analogs with a butyl (CMP-48), allyl (CMP-50), or phenyl ethyl (CMP-42) attached to an aromatic amine at the R2 position. None of these analogs showed any CCK1R activity. In addition, hydrazine (CMP-51), pyrrolidine CMP-64, or diethyl amine CMP-46, directly attached to the pyrimidine of the core, had significantly diminished responses as well. Furthermore, we evaluated derivatives connecting distinct side chains to the core via a sulfide bridge. The acetic acid analogs CMP-45 and CMP-59, the bromo butenyl (CMP-44), or the thiol (CMP-52) derivatives also displayed no effects. We also tested three compounds with N-substituted sulfanyl acetamides. Interestingly, the methoxy phenyl (CMP-40) and furanyl methyl (CMP-65) analogs, but not the bromo phenyl variant (CMP-41), showed strong activation of AVP2R-mediated cAMP accumulation; however, none of the sulfanyl acetamides showed any responses in the CCK1R IP-One PAM and agonist assays. Overall, we concluded that the CCK1R PAM activity likely relies on an ionic interaction of a negatively charged receptor residue with a basic amine attached to the tetracyclic core via a short alkyl linker important for correct positioning and orientation. These findings were further corroborated with 10 molecules containing a propyl or other alkyl side chain at the R1 position and non-basic functional groups as R2 substituents (Table 5), since none of these derivatives showed any significant activity in CCK1R IP-One assays. 

Hence, we proceeded to focus on analogs of the tetracyclic scaffold that incorporated a basic amino group at the R2 side chain. 

We were interested to see how much influence the oxygen or sulfur in the tetracyclic core has on the pharmacological profile (Table 6). We tested CMP-18, the sulfur analog of “hit 6”, with a propyl side chain at R1, a cyclohexyl ring A, and a dimethyl amine connected via a propyl linker to the aminopyrimidine of the core (R2). In CCK1R IP-One assays we found slightly elevated responses in the PAM format (score 2.0) compared to “hit 6”, while maintaining negligible activity in agonist mode. However, even though the response was attenuated, the sulfur derivative also maintained a significant off-target effect at AVP2Rs (score 2.0). We tested three further derivatives of “hit 6” incorporating oxygen in the core. The extension of the dimethyl amine to diethyl amine (CMP-36) appeared to attenuate agonist and PAM effects. Removing the hydroxyethyl to obtain the piperazine analog CMP-12, we were able to increase CCK1R IP-One PAM activity but also effects on AVP2R with activity scores of 2.2 and 3.3, respectively. In addition, we evaluated the propyl analog of “hit 1”, CMP-7, which contained a *N*-diethyl diamino ethyl at R2. We found improved CCK1R PAM activities (score 2.4) and attenuated off-target effects (score 1.6 AVP2R) compared to the *N*-dimethyl diamino propyl analog CMP-18. Exchange of the diethyl amino group into a morpholine (CMP-8) led to increased AVP2R activity (score 2.3). Truncation of the propyl at R1 to methyl (CMP-61) or extension to a butyl side chain (CMP-32) attenuated agonist and PAM activities. However, keeping the butyl side chain at R1, condensing ring A to a cyclopentane, and attaching a diamino propyl with a primary amino group at R2, we obtained analog CMP-5 with improved CCK1R PAM activities (score 2.7 (IP-One), non-significant intrinsic agonist activity, and minimal AVP2R off-target effects (score 1.6). 

Moreover, we studied 10 analogs with branched alkyl side chains at R1 (Table 7). The isopropyl analog of “hit 6”, CMP-24, with *N*-dimethyl diamino propyl at R2 and a cyclopentyl ring A, showed minimal activity in the IP-One PAM assay (score 1.8) and minimal off-target effects in AVP2Rs (score 1.6). Truncation to *N*-dimethyl diamino ethyl resulted in CMP-20, which showed a very similar profile with slightly increased PAM activity in CCK1R IP-One (score 1.9). Exchanging the dimethyl amine to a morpholine, oxygen to sulfur, and cyclopentyl to cyclohexyl (CMP-54), we found that the compound had an inverted selectivity profile, with no significant activity in CCK1R PAM format, while maintaining activity at AVP2Rs (score 2.0). Additionally, the direct analog of this compound but with an isobutyl sidechain at R1 (CMP-34) displayed the same AVP2R-selective profile. We investigated other isobutyl analogs with oxygen in the tetracyclic core, four of which contained a cyclopentyl ring A. The *N*-dimethyl diamino ethyl analog (CMP-21) showed similar activity in CCK1R IP-One PAM and AVP2R counter-screen assays (score 1.9 (CCK1R), 1.7 (AVP2R)). Substitution of the dimethyl amine to diethyl amine (CMP-33) resulted in attenuated responses. Elongation of the linker to diamino propyl (CMP-10) resulted in enhanced responses in both CCK1R IP-One PAM (score 2.3) and AVP2R cAMP (score 2.2) assays. Interestingly, the *N*-dimethyl diamino propyl analog (CMP-13) maintained CCK1R PAM activity in IP-One assays (score 2.2) and showed non-significant effects in the AVP2R counter-screen (score 1.4). In contrast, the cyclohexyl analogs CMP-25 and CMP-15, with a *N*-dimethyl diamino ethyl and piperazine group at R2, respectively, appeared to be AVP2R-selective (scores 3.0–3.8) with moderate CCK1R PAM activities (scores 1.8–2.0). 

Then, we evaluated seven analogs with a piperazine side chain at R1 (Table 8). CMP-6, the direct analog of “hit 1”, demonstrated robust CCK1R IP-One PAM activity (score 2.6) with minimal activity at AVP2Rs (score 1.7). Removal of the two ethyl groups from the basic amine resulted in the primary amine analog CMP-22, which showed a very different activity profile. CMP-22 maintained moderate CCK1R IP-One PAM activity (score 1.9). However, it demonstrated strong off-target activity (score 2.9 (AVP2R)) and substantial CCK1R intrinsic agonist activity (score 2.5 (IP-One). Interestingly, the dimethyl amino analog CMP-35 showed attenuated activities in CCK1R IP-One PAM (score 1.5) and AVP2R cAMP (score 1.6) assays. Moreover, changing the pyrimidine ring to a triazine within the tetracyclic core but keeping the *N*-dimethyl diamino ethyl group (CMP-27), we found the desired CCK1R PAM profile with an activity score of 1.7, and no intrinsic agonist activity. However, CMP-27 induced strong responses in AVP2R cAMP assays (score 3.5). Additionally, the triazine analog with a slightly elongated *N*-dimethyl diamino propyl moiety at R2 (CMP-23) demonstrated a similar profile, but substantial off-target activity (score 2.9 (AVP2R)). We also restrained the tertiary amine with an N-methyl piperazine analog containing the pyrimidine ring at the core (CMP-31), which resulted in weak PAM activities at CCK1Rs (scores 1.5 (CCK1R IP-One). As the last analog in this subset, we tested a diamino ethyl primary amine analog containing a methyl instead of a cyclohexyl ring (CMP-29). This modification led to increased agonist activity (score 2.3) over CCK1R PAM responses (score 1.6). 

Next, we characterized four analogs incorporating a phenyl side chain at R1. The direct analog of “hit 1” (CMP-11) with *N*-diethyl diamino ethyl function at R2 displayed an overall promising profile with good CCK1R PAM activity (scores 2.3), and no to minimal intrinsic agonist activities (scores 1.0). However, CMP-11 was not selective towards CCK1R with an AVP2R activity score of 2.4. Truncation of the ethyl groups to a dimethyl amine led to a compound (CMP-14) with a very similar CCK1R activity profile but that appeared to enhance the preference for AVP2Rs (score 3.0). Interestingly, incorporating a morpholine with reduced basicity as a tertiary amino function (CMP-26), we found a molecule with moderate CCK1R PAM activities (scores 1.8), no to minimal intrinsic agonist activity, and, moreover, negligible off-target effects (score 0.9 (AVP2R)). For structural variations, we also tested an analog with cyclopentyl ring A and an N-hydroxyethyl piperazine at R2 (CMP-37). This combination of residues resulted in a loss of activities across all formats. 

The final subset of analogs we investigated in more detail were “hit 1” derivatives containing a morpholine as side chain R1 (Table 9). “Hit 1” (CMP-1) demonstrated its highest activity in the CCK1R IP-One PAM primary screening assay (score 2.9), with no significant intrinsic agonist activity and minimal off-target effects (score 1.0 (AVP2R). Truncation of the diethyl to dimethyl amine (CMP-4) resulted in slightly higher AVP2R activity (score 1.2) with overall similar, but slightly attenuated responses in CCK1R assays. CMP-19, with a conformationally restrained N-methyl piperazine residue, R2, showed diminished signaling across all assays with modest effects in CCK1R IP-One PAM mode (score 1.9). Interestingly, removal of the methyl group from the piperazine resulted in analog CMP-3 with improved CCK1R IP-One activity (score 2.8) and minimal AVP2R off-target response (score 1.6). At this point, we also compared a piperazine analog with a diethyl amino group as R1 and a methyl instead of ring A (CMP-16). This overall truncated analog showed attenuated responses across all formats with a CCK1R IP-One PAM activity score of 2.0. CMP-17, the triazine analog with cyclohexyl ring A and a *N*-dimethyl diamino propyl side chain at R2, displayed a weak but overall promising pharmacological profile with modest CCK1R PAM activity (scores 2.0), no intrinsic agonist activity, and minimal AVP2R off-target effects (score 1.2). However, the pyrimidine analog with *N*-dimethyl diamino propyl at R2 and a cyclopentyl ring A (CMP-43) showed no agonist or PAM activity. We found analogs with a dimethyl thiacyclohexyl ring A, which we thought would expand the molecule and add bulkiness to the cyclohexyl moiety. CMP-2, the direct analog to “hit 1” with an *N*-diethyl diamino ethyl function at R2, displayed a very promising profile with robust CCK1R IP-One PAM activity (score 2.8) and a non-significant agonist response. Moreover, the introduction of the dimethyl thiacyclohexyl moiety eradicated responses in AVP2R cAMP assays. Additionally, the truncation of the diethyl to a dimethyl amino group (CMP-9), which tended to enhance AVP2R signaling for other molecules, maintained strong CCK1R-selectivity (score 2.3) by carrying the dimethyl thiacyclohexyl ring A. 

**Figure 7:**
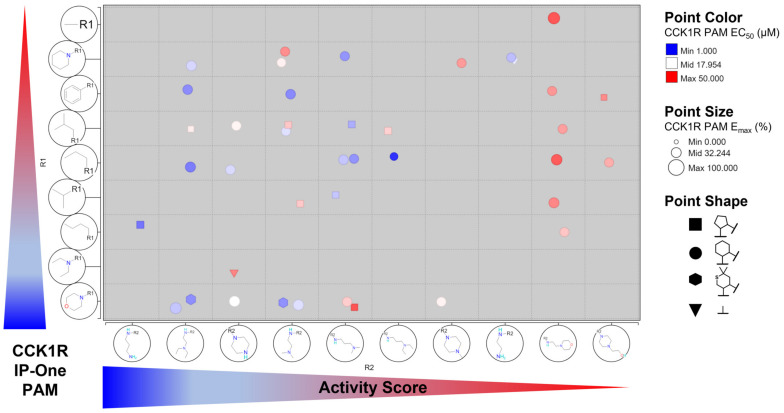
R-group analysis of 40 tetracyclic analogs with basic amino functions. Compounds identified by R1, R2, and point shape (ring A); point color indicates CCK1R PAM EC_50_ (µM), point size indicates CCK1R PAM E_max_ (%); R1 and R2 groups ranked via median activity score with higher scores at bottom left corner. R-group analysis was generated via the Certara D360 scientific informatics platform.

To evaluate more globally how distinct modifications influence CCK1R PAM activity, we performed an R-group analysis (Figure 7). We looked at the 40 analogs containing a basic amino function and grouped them based on their R1 and R2 side chains, whereas the distinct R2 modifications were plotted on the *x*-axis and the different R1 side chains were plotted on the *y*-axis. The order of residues was determined by the CCK1R PAM activity score as a median value for each group with the same R1 or R2 residues, ranking the best modifications at the bottom-left corner. The R-group analysis confirmed that the morpholine at R1 and a *N*-diethyl diamino ethyl side chain as R2 seemed to generally enhance the activities of the tetracyclic scaffold. CMP-5, which is constituted by a butyl side chain at R1 and a diamino propyl with a primary amino group at R2, is only a single data point and is therefore difficult to compare with other residues. However, it could indicate a potentially promising direction for a future targeted medicinal chemistry effort. Next, we wanted to verify that the activity score reflects potencies and efficacies of the analogs. Therefore, we colored points, representing a distinct molecule, based on their potencies (red—white—blue, with blue being best potency), and modified the point sizes according to their efficacies (the bigger the more efficacious). We found that red-colored compounds were largely found at the right-top, whereas blue-colored molecules with larger points had tendencies towards the bottom-left corner, indicating that the activity score is a good tool to rank order compounds and residues.

Upon CCK binding, the CCK1R preferably coupled to Gq/11 proteins; however, at higher concentrations of CCK, CCK1R-activated Gs signaling was observed as well. Therefore, we investigated our original hits and our best analogs incorporating the dimethyl thiacyclohexyl ring at R1 for their ability to enhance CCK-mediated cAMP accumulation in HEK-293 cells overexpressing CCK1R. We utilized an EC_20_ concentration of CCK for basal stimulation and found that “hit 1” (EC_50_ 14.7 ± 7.3 μM, E_max_ 48 ± 31%; *n* = 8) and “hit 6” (EC_50_ 15.6 ± 12.4 μM, E_max_ 56 ± 26%; *n* = 8) showed comparable PAM activity in cAMP as in IP-One assays. We further evaluated CCK1R-selective dimethyl thiacyclohexyl analogs CMP-2 (EC_50_ 6.0 ± 0.9 μM, E_max_ 28 ± 3%; *n* = 2) and CMP-9 (EC_50_ 5.9 ± 0.1 μM, E_max_ 30 ± 1%; *n* = 2), which demonstrated slightly improved potencies but attenuated efficacies compared to the original hits, in alignment with their activities in IP-One PAM assays. Hence, this tetracyclic CCK1R PAM scaffold seems to universally enhance CCK-mediated CCK1R signaling without discriminating between the distinct G protein pathways.

## 4. Discussion

Obesity is a major health problem around the world, with ongoing clinical need for new effective management strategies [13,14,15,16,17]. While CCK1R was long ago identified as a potential target for such therapy [1,18], full agonists of this receptor failed to meet primary end points in clinical trials, since they were no more effective than acute dieting to induce weight loss [19,20,21,22]. There was hesitance in preparing more potent and longer duration agonists, which were expected to have pronounced side effects and potential on-target toxicity [23,24,25]. However, we proposed a distinct strategy to utilize PAMs [26] of CCK action at CCK1R that possessed minimal or no intrinsic agonist activity [2,3,5]. Such an agent would be expected to enhance the satiety effect of endogenously released CCK during and after a meal to reduce meal size in a temporally finite manner. There was also the additional benefit that such an agent could reverse the negative impact of elevated membrane cholesterol on stimulus–activity coupling at this receptor [27,28]. 

We recently described our effort to identify candidates with this pharmacologic profile in a high throughput screening effort targeting small molecules [6]. Characterization and optimization of the best candidate scaffold identified in that work is the focus of the current report. This is a tetracyclic scaffold represented by “hit 1” and “hit 6” from the earlier report [6]. Here, we extensively characterize these candidates and demonstrate that they possess all the pharmacologic characteristics being sought. This includes the ability to enhance CCK action both in normal healthy cells and those with increased membrane cholesterol. They also exhibit minimal intrinsic agonist action under either condition. Under conditions in which they behave as PAMs, they do not induce CCK1R internalization, thereby priming this receptor to enhance the activity of endogenous released hormone at a temporally appropriate time. 

We further conducted comprehensive structure–activity relationship studies to elucidate structural determinants for developing a PAM without intrinsic agonist activity. Therefore, we acquired 63 commercially available analogs of the original hits. We determined an activity score to facilitate the comparison and ranking of analogs that might be dependent on impact on potency and/or efficacy to exert positive allosteric effects. A previous study [29] proposed the calculation of R_max_/R_50_ or ΔLog(R_max_/R_50_) as a system-independent scale of relative activity, which also includes both parameters, potency and efficacy, of a compound dose–response determined in a PAM screening assay. We calculated both the activity scores and Log(R_max_/R_50_) values for our CCK1R PAMs and found that Log(R_max_/R_50_) relies excessively on the potency of a compound rather than also considering its efficacy. For example, for our most promising analogs in the CCK1R IP-One PAM assay, “hit 1” (EC_50_ 12.8 μM, E_max_ 60%) and CMP-2 (EC_50_ 9.2 μM, E_max_ 57%), we calculated Log(R_max_/R_50_) values of 4.7 and 4.8 compared to activity scores of 2.9 and 2.8, respectively. Hence, in contrast to utilizing the activity score, CMP-2 would display a slightly higher PAM activity compared to “hit 1” using Log(R_max_/R_50_). However, for an analog with minimal efficacy, such as the non-basic molecule CMP-38 (EC_50_ 7.6 μM, E_max_ 20%), we assessed a Log(R_max_/R_50_) of 4.4 and activity score of 1.0. Hence, in this case the Log(R_max_/R_50_) would have overstated the strength of CMP-38, whereas the activity score displayed it as a molecule with non-significant activity. We believe that the Log(R_max_/R_50_) might be superior for smaller sets of analogs with overall strong effects, while we found that the activity score worked better as a tool for SAR of larger sets of analogs derived from an HTS campaign that often start with moderate potency and efficacy. 

One aim of this study was to further investigate the scaffolds we had identified in our HTS campaign [6] using the analog-by-catalog approach. Our target molecule would need to meet four criteria to display the desired pharmacological profile and justify expanded medicinal chemistry approaches. Firstly, the target molecule should display substantial positive modulation of CCK-mediated CCK1R Gq-signaling. Secondly, the desired compound should display no to minimal intrinsic agonist activity at CCK1Rs. As a third criterion, the molecule should maintain its CCK1R PAM profile in an environment with elevated cholesterol. As the fourth critical point, the compound should be selective toward CCK1Rs. 

Initially, we tested our original “hits” having the tetracyclic scaffold and found that both displayed the desired profile of a CCK1R PAM with minimal intrinsic agonist activity [6], and moreover, both compounds maintained their PAM effects at CCK1R-Y140A cells [11] or in a system with excess cholesterol [10] (Figure 3). However, the original hits showed significant off-target effects at AVP2Rs [6]. Hence, one of our main goals for this study was to investigate structural modifications that would be able to eliminate the undesired AVP2R activity, while maintaining or enhancing clean PAM effects at CCK1R-WT.

Using the activity score as a ranking tool for PAMs, we were able to identify structural components contributing to distinct pharmacological effects. After comprehensive SAR analysis of 65 commercially available analogs, we observed the following overall trends: (i) A basic amino function at the R2 position is required for CCK1R PAM activity, but not necessarily for AVP2R signaling. (ii) Most of the tested analogs displayed no or insignificant intrinsic agonist activity in the CCK1R IP-One format, except analogs with a combination of a primary diamino ethyl group at R2 and a piperidine moiety at R1 (CMP-22, CMP-29). (iii) In general, analogs with an *N*-diethyl diamino ethyl side chain at R2 showed reduced AVP2R off-target effects compared to direct analogs with *N*-dimethyl diamino ethyl groups. This could indicate steric clashes with AVP2Rs at this position. (iv) In addition, a direct comparison of a sulfur (CMP-18) and oxygen (“hit 6”) (CMP-28) analog indicated enhanced CCK1R PAM activity with reduced AVP2R signaling for CMP-18. 

Overall, cyclohexyl analogs at ring A showed more promising profiles than methyl or cyclopentyl derivatives, except CMP-5, which carries a butyl sidechain at R1 and a diamino propyl function at R2. CMP-5 displayed substantial CCK1R PAM activity in IP-One (score 2.7) assays with minimal but still significant AVP2R activation (score 1.6). Thus, it is likely a combination of distinct structural components that leads to the required positioning and steric hindrance to mediate the desired selectivity. 

A significant finding was that the substitution of the cyclohexyl ring to a dimethyl thiacyclohexyl moiety was able to completely eradicate AVP2R effects while maintaining CCK1R PAM activity in the IP-One format. This substantiated our hypothesis that slight structural expansion could induce steric clashes at AVP2Rs but not in the allosteric pocket of CCK1Rs. 

Hence, we were able to demonstrate that structural modifications can eliminate unwanted activity at AVP2Rs but found limitations using the analog-by-catalog approach. We think that specific combinations, such as incorporating a dimethyl thiacyclohexyl ring and attaching specific residues suggested by the R-group analysis (Figure 6 and Figure 7), would be excellent starting points for a planned medicinal chemistry effort. Therefore, we believe that the insights gained in this study will greatly improve our future hit-to-lead campaign using targeted organic synthesis of analogs or computational docking of PAMs into the recently solved cryo-EM structure of CCK1R [30].

## Figures and Tables

**Table 1:** Agonist-induced intracellular calcium responses in CHO cell lines in the absence and presence of PAM compounds.

Receptor-Ligands	pEC_50_	*n*, *p* Values
**CCK1R**		
CCK-8	10.6 ± 0.1	5
CCK-8 + “hit 1”	11.1 ± 0.1 *	5, 0.048
CCK-8 + “hit 6”	11.5 ± 0.2 **	5, 0.002
**CCK1R**		
CCK-33	9.6 ± 0.2	5
CCK-33 + “hit 1”	10.7 ± 0.2 *	5, 0.016
CCK-33 + “hit 6”	10.7 ± 0.2 **	5, 0.008
**CCK2R**		
CCK-8	9.3 ± 0.1	6
CCK-8 + “hit 1”	9.5 ± 0.1	6, 0.20
CCK-8 + “hit 6”	9.5 ± 0.1	6, 0.19
**CCK1R(Y140A)**		
CCK-8	9.0 ± 0.1	5
CCK-8 + “hit 1”	9.7 ± 0.1 ***	5, 0.0008
CCK-8 + “hit 6”	10 ± 0.1 ***	5, <0.0001
**CCK1R**		
CCK-OPE	7.3 ± 0.2	5
CCK-OPE + “hit 1”	8.0 ± 0.2	5, 0.08
CCK-OPE + “hit 6”	8.1 ± 0.2 *	6, 0.04
**CCK1R + excess cholesterol**		
CCK-8	9.4 ± 0.2	5
CCK-8 + “hit 1”	10.2 ± 0.2 **	5, 0.005
CCK-8 + “hit 6”	10.2 ± 0.2 **	6, 0.005

Values are expressed as means ± SEM of “*n*” independent experiments performed in duplicate. “Hits” 1 and 6 were utilized in 10 µM concentrations. Differences between control and the presence of “hit 1” and “hit 6” were determined using one-way ANOVA. * *p* < 0.05; ** *p* < 0.01; *** *p* < 0.001.

**Table 2:** Kinetic parameters for CCK binding to CCK1R-expressing membranes from CHO-CCK1R cells in the absence or presence of tetracyclic compounds.

	CCK-8	*n*	CCK-8 + “Hit 1”	*n*, *p* Values	CCK-8 + “Hit 6”	*n*, *p* Values
K_on_ rate, × 10^8^ M^−1^ min^−1^	0.7 ± 0.1	5	2.6 ± 0.6 *	3, 0.04	0.7 ± 0.4	5, 0.31
K_off_ rate, min^−1^	1.1 ± 0.1	5	0.4 ± 0.1 *	3, 0.04	0.5 ± 0.1 **	5, 0.01
pK_i_	7.8 ± 0.1	5	8.8 ± 0.1 *	3, 0.04	8.1 ± 0.2	5, 0.06

Values are expressed as means ± SEM from “*n*” independent experiments performed in triplicate. “Hits” 1 and 6 were utilized in 10 µM concentrations. Levels of significance for differences relative to CCK-8 controls were calculated using the Mann-Whitney test. * *p* < 0.05, ** *p* < 0.01.

**Table 3:** Cooperativity analysis of the impact of tetracyclic compounds on CCK-stimulated intracellular calcium responses in CCK1R-expressing cells.

	CCK-8 + “Hit 1”	CCK-8 + “Hit 6”
pK_b_	4.5 ± 0.4	4.6 ± 0.2
Tau K_b_	0.5 ± 0.1	0.4 ± 0.1
Logαβ	1.5 ± 0.3	1.0 ± 0.2
*n*	5	5

Values are expressed as means ± SEM from five experiments performed in duplicate. “Hits” 1 and 6 were utilized in 10 µM concentrations.

**Table 4:** Structure–activity studies of 15 morpholine analogs with non-basic amine R2 substitutions.

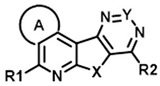														
ID	Structure					CCK1R PAM (IP-One)			CCK1R Ago (IP-One)			AVP2R PAM (cAMP)		
	R1	R2	A	X	Y	EC_50_ (µM)	E_max_ (%)	Score	EC_50_ (µM)	E_max_ (%)	Score	EC_50_ (µM)	E_max_ (%)	Score
CMP-40	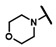	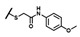	cyclohexyl	S	C	0	0	0	0	0	0	2.9 ± 0.1	96 ± 9	5.33
CMP-41	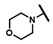	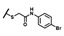	cyclohexyl	S	C	0	0	0	0	0	0	0	0	0
CMP-65	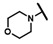	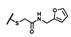	cyclohexyl	S	C	0	0	0	0	0	0	10.0 ± 1.7	96 ± 5	4.78
CMP-42	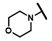	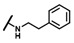	cyclohexyl	S	C	0	0	0	0	0	0	0	0	0
CMP-44	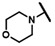	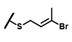	cyclohexyl	S	C	0	0	0	0	0	0	0	0	0
CMP-45	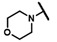	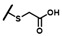	cyclohexyl	S	C	0	0	0	0	0	0	37.9 ± 7.0	33 ± 13	1.46
CMP-64	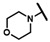	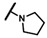	cyclohexyl	S	C	N.D.	N.D.	N.D.	0	0	0	0	0	0
CMP-46	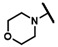	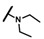	cyclohexyl	S	C	0	0	0	0	0	0	0	0	0
CMP-47	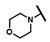	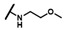	cyclohexyl	S	C	0	0	0	0	0	0	0	0	0
CMP-48	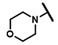	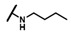	cyclohexyl	S	C	0	0	0	0	0	0	0	0	0
CMP-49	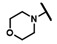	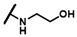	cyclohexyl	S	C	0	0	0	0	0	0	0	0	0
CMP-50	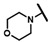	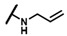	cyclohexyl	S	C	0	0	0	0	0	0	0	0	0
CMP-51	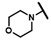	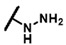	cyclohexyl	S	C	0	0	0	0	0	0	0	0	0
CMP-52	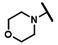	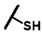	cyclohexyl	S	N	0	0	0	0	0	0	N.D.	N.D.	N.D.
CMP-59	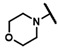	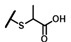	cyclohexyl	S	C	N.D.	N.D.	N.D.	0	0	0	22.3 ± 7.2	20 ± 0	0.93

Average potencies (EC_50_ (µM) ± standard deviation (SD)), average compound efficacy (E_max_ (%) ± SD), and calculated activity score (E_max_ × pEC_50_) of compound dose–response studies in screening mode. Compounds were tested in Cisbio IP-One and cAMP accumulation assays; N.D. = not determinable (response less than mean ± 3 × SD of negative control).

**Table 5:** Structure–activity studies of 10 alkyl analogs with non-basic amine R2 substitutions.

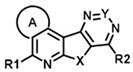														
ID	Structure					CCK1R PAM (IP-One)			CCK1R Ago (IP-One)			AVP2R PAM (cAMP)		
	R1	R2	A	X	Y	EC_50_ (µM)	E_max_ (%)	Score	EC_50_ (µM)	E_max_ (%)	Score	EC_50_ (µM)	E_max_ (%)	Score
CMP-56	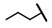	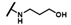	cyclopentyl	S	C	N.D.	N.D.	N.D.	0	0	0	0	0	0
CMP-57	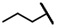	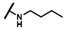	cyclohexyl	S	C	N.D.	N.D.	N.D.	0	0	0	0	0	0
CMP-58	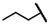	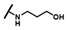	cyclohexyl	O	C	N.D.	N.D.	N.D.	0	0	0	0	0	0
CMP-60	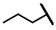	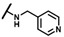	cyclohexyl	O	C	N.D.	N.D.	N.D.	0	0	0	0	0	0
CMP-38	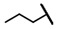	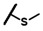	cyclohexyl	S	C	7.6 ± 3.3	20 ± 7.6	1.01	0	0	0	0	0	0
CMP-62	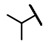	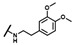	cyclopentyl	O	C	N.D.	N.D.	N.D.	0	0	0	0	0	0
CMP-39	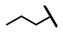	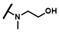	cyclohexyl	O	C	N.D.	N.D.	N.D.	0	0	0	0	0	0
CMP-63	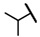	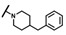	cyclopentyl	O	C	N.D.	N.D.	N.D.	0	0	0	0	0	0
CMP-55	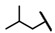	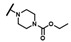	cyclohexyl	O	C	0	0	0	0	0	0	0	0	0
CMP-53	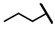	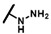	cyclohexyl	O	C	0	0	0	0	0	0	15.3 ± 9.8	61 ± 12	2.95

Average potencies (EC_50_ (µM) ± standard deviation (SD)), average compound efficacy (E_max_ (%) ± SD), and calculated activity score (E_max_ × pEC_50_) of compound dose–response studies in screening mode. Compounds were tested in Cisbio IP-One and cAMP accumulation assays; N.D. = not determinable (response less than mean ± 3 × SD of negative control).

**Table 6:** Structure–activity studies of 10 *n*-alkyl analogs.

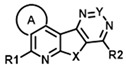														
ID	Structure					CCK1R PAM (IP-One)			CCK1R ago (IP-One)			AVP2R PAM (cAMP)		
	R1	R2	A	X	Y	EC_50_ (µM)	E_max_ (%)	Score	EC_50_ (µM)	E_max_ (%)	Score	EC_50_ (µM)	E_max_ (%)	Score
CMP-5	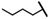	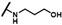	cyclopentyl	S	C	7.5 ± 2.9	53 ± 6	2.66	N.D.	N.D.	N.D.	8.1 ± 8.5	32 ± 11	1.61
CMP-7	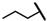	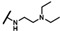	cyclohexyl	S	C	8.0 ± 2.1	48 ± 11	2.39	0	0	0	6.5 ± 4.2	31 ± 2	1.61
CMP-8	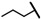	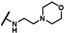	cyclohexyl	S	C	37.8 ± 3.9	53 ± 1	2.34	0	0	0	15.7 ± 8.4	48 ± 12	2.31
CMP-12	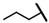	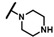	cyclohexyl	O	C	14.1 ± 4.3	46 ± 7	2.23	N.D.	N.D.	N.D.	11.4 ± 3.6	66 ± 3	3.28
CMP-18	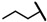	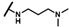	cyclohexyl	S	C	12.7 ± 5.5	43 ± 21	1.96	0	0	0	11.8 ± 14.0	40 ± 17	1.99
CMP-28 (“hit 6”)	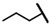	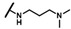	cyclohexyl	O	C	9.5 ± 3.5	35 ± 13	1.66	N.D.	N.D.	N.D.	15.0 ± 13.6	73 ± 16	3.53
CMP-30	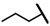	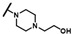	cyclohexyl	O	C	27.1 ± 10.7	36 ± 16	1.55	0	0	0	5.9 ± 3.8	29 ± 0	1.52
CMP-32	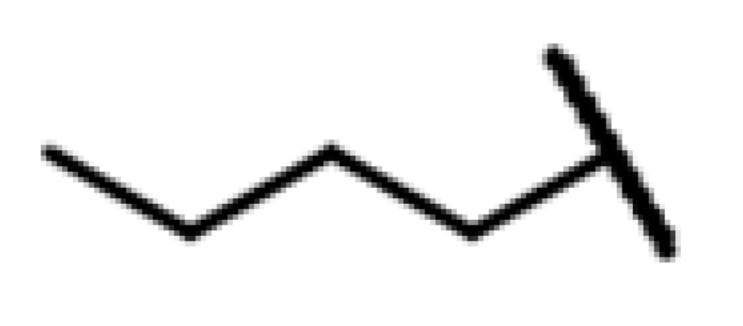	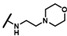	cyclohexyl	S	C	23.9 ± 25.8	41 ± 34	1.52	0	0	0	9.3 ± 11.1	33 ± 26	1.67
CMP-36	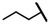	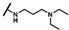	cyclohexyl	O	C	3.3 ± 5.4	25 ± 10	1.32	0	0	0	6.1 ± 2.8	27 ± 20	1.41
CMP-61	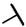	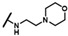	cyclohexyl	S	C	N.D.	N.D.	N.D.	0	0	0	0	0	0

Average potencies (EC_50_ (µM) ± standard deviation (SD)), average compound efficacy (E_max_ (%) ± SD), and calculated activity score (E_max_ × pEC_50_) of compound dose–response studies in screening mode. Compounds were tested in Cisbio IP-One and cAMP accumulation assays; N.D. = not determinable (response less than mean ± 3 × SD of negative control).

**Table 7:** Structure–activity studies of 10 branched alkyl analogs.

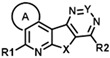														
ID	Structure					CCK1R PAM (IP-One)			CCK1R ago (IP-One)			AVP2R PAM (cAMP)		
	R1	R2	A	X	Y	EC_50_ (µM)	E_max_ (%)	Score	EC_50_ (µM)	E_max_ (%)	Score	EC_50_ (µM)	E_max_ (%)	Score
CMP-10	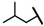	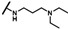	cyclopentyl	O	C	22.5 ± 9.0	51 ± 16	2.28	N.D.	N.D.	N.D.	21.7 ± 11.3	46 ± 9	2.15
CMP-13	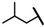	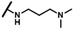	cyclopentyl	O	C	11.2 ± 4.2	52 ± 36	2.23	0	0	0	12.6 ± 7.9	28 ± 14	1.35
CMP-15	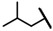	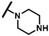	cyclohexyl	O	C	17.8 ± 6.2	43 ± 3	2.04	0	0	0	7.3 ± 3.3	58 ± 15	2.97
CMP-20	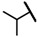	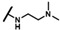	cyclopentyl	O	C	23.6 ± 1.0	42 ± 12	1.90	0	0	0	21.2 ± 19.7	25 ± 1	1.17
CMP-21	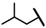	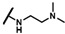	cyclopentyl	O	C	23.8 ± 10.5	45 ± 23	1.90	0	0	0	22.8 ± 3.8	37 ± 4	1.74
CMP-24	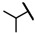	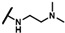	cyclopentyl	O	C	12.7 ± 15.5	48 ± 45	1.81	0	0	0	19.6 ± 5.1	34 ± 23	1.62
CMP-25	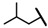	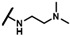	cyclohexyl	O	C	14.5 ± 5.3	38 ± 11	1.79	N.D.	N.D.	N.D.	8.6 ± 5.4	74 ± 12	3.75
CMP-33	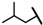	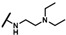	cyclopentyl	O	C	18.8 ± 7.7	35 ± 19	1.51	0	0	0	21.0 ± 6.5	34 ± 1	1.60
CMP-34	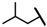	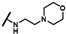	cyclohexyl	S	C	29.0 ± 13.1	34 ± 13	1.50	0	0	0	7.8 ± 4.0	42 ± 17	2.13
CMP-54	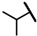	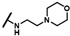	cyclohexyl	S	C	N.D.	N.D.	N.D.	0	0	0	26.2 ± 2.4	43 ± 7	1.96

Average potencies (EC_50_ (µM) ± standard deviation (SD)), average compound efficacy (E_max_ (%) ± SD), and calculated activity score (E_max_ × pEC_50_) of compound dose–response studies in screening mode. Compounds were tested in Cisbio IP-One and cAMP accumulation assays; N.D. = not determinable (response less than mean ± 3 × SD of negative control).

**Table 8:** Structure–activity studies of seven piperazine and four phenyl analogs.

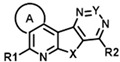														
ID	Structure					CCK1R PAM (IP-One)			CCK1R ago (IP-One)			AVP2R PAM (cAMP)		
	R1	R2	A	X	Y	EC_50_ (µM)	E_max_ (%)	Score	EC_50_ (µM)	E_max_ (%)	Score	EC_50_ (µM)	E_max_ (%)	Score
CMP-6	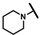	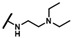	cyclohexyl	S	C	13.8 ± 3.2	57 ± 27	2.58	0	0	0	9.9 ± 0.5	33 ± 1	1.67
CMP-11	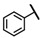	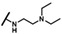	cyclohexyl	S	C	9.1 ± 4.8	45 ± 6	2.27	27.1 ± 15.3	21 ± 2	0.96	3.2 ± 1.1	44 ± 8	2.44
CMP-14	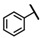	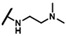	cyclohexyl	S	C	8.9 ± 1.7	44 ± 10	2.17	29.7 ± 8.0	17 ± 5	0.72	4.7 ± 4.1	56 ± 33	2.97
CMP-22	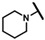	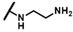	cyclohexyl	S	C	12.2 ± 4.6	39 ± 11	1.87	26.9 ± 1.1	60 ± 4.5	2.76	2.7 ± 0.7	52 ± 4	2.92
CMP-23	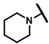	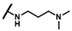	cyclohexyl	S	N	9.6 ± 3.6	38 ± 13	1.86	30.0 ± 4.9	41 ± 1	1.85	3.1 ± 1.3	53 ± 3	2.93
CMP-26	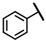	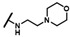	cyclohexyl	S	C	30.2 ± 11.2	41 ± 15	1.76	N.D.	N.D.	N.D.	3.7 ± 0.5	16 ± 4	0.87
CMP-27	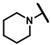	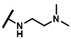	cyclohexyl	S	N	18.4 ± 8.1	38 ± 14	1.70	0	0	0	9.1 ± 1.0	70 ± 4	3.51
CMP-29	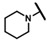	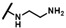	4-methyl	S	C	15.1 ± 1.8	33 ± 7	1.59	52.9 ± 39	39 ± 12	1.67	19.2 ± 0.8	35 ± 0	1.66
CMP-31	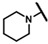	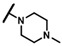	cyclohexyl	S	C	29.6 ± 1.8	35 ± 14	1.54	0	0	0	5.4 ± 1.7	24 ± 3	1.26
CMP-35	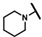	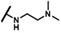	cyclohexyl	S	C	31.3 ± 14.9	36 ± 20	1.49	0	0	0	7.9 ± 2.5	31 ± 0	1.59
CMP-37	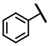	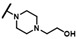	cyclopentyl	S	C	31.7 ± 1.2	28 ± 9	1.26	0	0	0	0	0	0

Average potencies (EC_50_ (µM) ± standard deviation (SD)), average compound efficacy (E_max_ (%) ± SD), and calculated activity score (E_max_ × pEC_50_) of compound dose–response studies in screening mode. Compounds were tested in Cisbio IP-One and cAMP accumulation assays; N.D. = not determinable (response less than mean ± 3 × SD of negative control).

**Table 9:** Structure–activity studies of eight morpholine and one *N*-diethyl analogs.

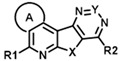														
ID	Structure					CCK1R PAM (IP-One)			CCK1R Ago (IP-One)			AVP2R PAM (cAMP)		
	R1	R2	A	X	Y	EC_50_ (µM)	E_max_ (%)	Score	EC_50_ (µM)	E_max_ (%)	Score	EC_50_ (µM)	E_max_ (%)	Score
CMP-1 (“hit 1”)	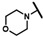	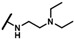	cyclohexyl	S	C	12.8 ± 4.6	63 ± 18	2.94	N.D.	N.D.	N.D.	12.0 ± 7.1	20.7 ± 9.8	1.02
CMP-2	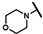	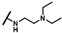	dimethyl thiacyclohe-xyl	S	C	9.2 ± 2.0	57 ± 12	2.82	31.3 ± 3.1	31 ± 15	1.31	N.D.	N.D.	N.D.
CMP-3	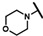	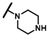	cyclohexyl	S	C	16.6 ± 15.9	60 ± 23	2.77	N.D.	N.D.	N.D.	14.8 ± 10.3	34.0 ± 5.3	1.64
CMP-4	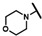	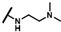	cyclohexyl	S	C	14.4 ± 1.5	62 ± 33	2.71	0	0	0	12.8 ± 14.0	24.5 ± 7.1	1.20
CMP-9	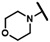	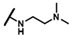	dimethyl thiacyclohe-xyl	S	C	9.3 ± 1.1	46 ± 3	2.31	N.D.	N.D.	N.D.	N.D.	N.D.	N.D.
CMP-16	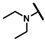	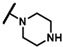	methyl	S	C	32.6 ± 12.5	45 ± 7	2.02	0	0	0	7.6 ± 8.2	17 ± 4	0.87
CMP-17	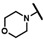	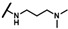	cyclohexyl	S	N	23.0 ± 17.9	50 ± 32	1.99	N.D.	N.D.	N.D.	22.4 ± 9.2	26 ± 1	1.20
CMP-19	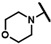	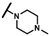	cyclohexyl	S	C	18.2 ± 8.6	51 ± 43	1.94	0	0	0	N.D.	N.D.	N.D.
CMP-43	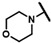	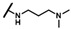	cyclopentyl	S	C	N.D.	N.D.	N.D.	0	0	0	N.D.	N.D.	N.D.

Average potencies (EC_50_ (µM) ± standard deviation (SD)), average compound efficacy (E_max_ (%) ± SD), and calculated activity score (E_max_ × pEC_50_) of compound dose–response studies in screening mode. Compounds were tested in Cisbio IP-One and cAMP accumulation assays; N.D. = not determinable (response less than mean ± 3 × SD of negative control).

## Data Availability

The data presented in this study are available upon request from the corresponding author.
